# Older adults’ and service providers’ experiences of a settings-based health promotion initiative in English football

**DOI:** 10.1093/heapro/daad027

**Published:** 2023-06-20

**Authors:** Patricia C Jackman, Aoife Lane, Jacquelyn Allen-Collinson, Hannah Henderson

**Affiliations:** School of Sport and Exercise Science, University of Lincoln, Lincoln, UK; Department of Sport and Health Sciences, Technological University of the Shannon, Athlone, Ireland; School of Sport and Exercise Science, University of Lincoln, Lincoln, UK; School of Sport and Exercise Science, University of Lincoln, Lincoln, UK

**Keywords:** physical activity, sports club, community, ageing

## Abstract

The study was undertaken to explore the experiences of older adults and service providers in a settings-based health promotion initiative in a football club. We conducted semi-structured interviews with 10 older adults attending an ‘Extra Time Hub’ (ETH) and two staff delivering the initiative. Our reflexive thematic analysis generated six themes. Findings revealed the brand of the sports club attracted some to join the ETH, but through partnerships with local agencies, the initiative was also successful in widening participation beyond older adults with an interest in football. Participants perceived that the ETH was beneficial for their mental health, helped them develop social connections, and promoted positive physical activity experiences. Moreover, the variety of pleasures derived from participation were also discussed. Our findings also illustrate the central role of staff in older adults’ experiences of this form of health promotion. Overall, this study contributes to understandings of settings-based health promotion activities in sports clubs, and also demonstrates the potential for sports clubs to widen engagement with the local community through health promotion for older adults.

## Graphical Abstract

**Graphical Abstract:**
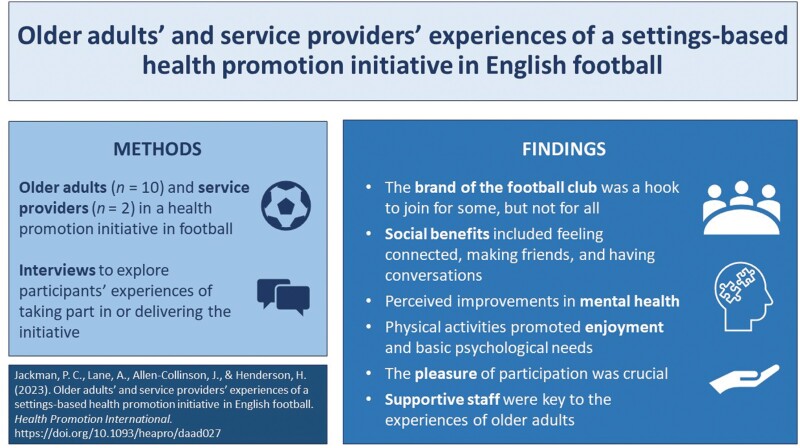

## INTRODUCTION

Promoting health and wellbeing as people age has been highlighted as a global priority (World Health Assembly, 2020). One approach to promote health and wellbeing in older adulthood is frequent physical activity (National Institute on Aging, 2022). Being physically active in later life reduces the risk of cardiovascular disease (Soares-Miranda *et al*., 2016), osteoporosis (McMillan *et al*., 2017), cognitive impairment (Zhu *et al*., 2017) and mental ill-health (Cunningham *et al*., 2020). Furthermore, physical activity in older adulthood can promote pleasure and a sense of wellbeing (Allen-Collinson *et al*., 2011; Phoenix and Orr, 2014; Williams *et al*., 2018) and facilitate social interactions (Franke *et al*., 2021). To improve older adults’ wellbeing and independence, service providers, practitioners and commissioners have been advised to establish initiatives to help older adults build or sustain social participation and engage in tailored, community-based physical activity (National Institute for Health and Care Excellence, 2015; Wolter *et al*., 2021). In this article, we report on a qualitative research project that explored older adults’ and service providers’ perspectives on a community-based, physical activity health promotion initiative delivered within a sports club; a setting increasingly being used to promote physical activity and health across the lifespan.

### Settings-based approach to health promotion

Settings are places where people live, learn, work and play, and where their health may be impacted by environmental, social, organizational and personal factors. Engaging people with health promotion in settings and making these contexts more supportive of health is one of the original and lasting principles of health promotion, as defined in the Ottawa Charter (World Health Organization [WHO], 1986). Subsequently, cities, schools and workplaces have become established contexts for implementing a settings-based approach to health promotion, typically adopting individually-oriented interventions rather than targeting change to the broader setting itself (Newman *et al*., 2015; Kokko and Baybutt, 2022). This individually-oriented approach reflects a ‘passive’, ‘active’ or ‘vehicle’ model, where the setting offers access to participants for various health promotion initiatives, while also striving to provide some internal education and policy change around specific health topics. In contrast, an ‘organic’ or ‘comprehensive’ approach involves alignment of the core business of the setting with health and, as a result, seeks changes to the setting culture and structure (Whitelaw *et al*., 2001). Here, settings are systems with actions underpinned by a socio-ecological approach to health (Dooris, 2004) that should extend beyond the system itself to consider broader collaborations, relationships and interconnectedness (Rutter *et al*., 2018).

In the early 2000s, the sports club was recognized as a setting where the whole system of the club and external actors could impact positively on the physical, mental and social health of members (Van Hoye *et al*., 2022). A health-promoting sports club (HPSC) model has since been established and refined to support implementation of HPSC activities. These may be targeted to address cultural (e.g. club policy), environmental (e.g. facilities, expertise), social (e.g. attitudes to sport and health promotion) and economic (e.g. time and money) determinants of health in the club, and delivered across all levels, from club policy to coach–player interactions (Kokko *et al*., 2013; Johnson *et al*., 2019; Van Hoye *et al*., 2020). There have been many subsequent efforts to understand, describe and deliver health promotion in sports clubs in Europe and worldwide (Kokko *et al*., 2016). In turn, Geidne and Van Hoye (2021) differentiated between types of health promotion activity in sports clubs, suggesting that these can range from communicating the health benefits of participation in sport through to using sports settings to deliver health promotion activities aimed at specific groups. Such health promotion initiatives are accessible and feasible for most sports clubs, while some can evolve to a HPSC, which involves the sport club system embracing health promotion in all of its activity. This is more complex and challenging to deliver, requiring commitment and investment by national sporting organizations (Lane *et al*., 2021). Van Hoye *et al.* proposed that indicators of a HPSC include the promotion of health beyond physical activity or a singular topic, engagement with internal and external partners, and establishing a long-term commitment to health promotion (Van Hoye *et al*., 2021). Recent reviews of health promotion interventions in sports clubs (McFadyen *et al*., 2018; Geidne *et al*., 2019) have indicated that this activity is continuously growing. Despite this, evidence of the health impacts for any type of health promotion activity in sports clubs is lacking, largely due to poor research design and evaluation, alongside few examples of HPSCs.

#### Health promotion in football clubs

Football (in this article, we use the term ‘football’ to refer to the sport of soccer, as is customary in the UK) is one of the most popular sports in the world and, therefore, it is unsurprising that a football setting was found to be the most used sport context for health promotion (Geidne *et al*., 2019). Programmes have focussed on healthy stadium environments (Drygas *et al*., 2011; Van Hoye *et al*., 2022), coach and youth player health education (Fuller *et al*., 2016), player welfare (Sadigursky *et al*., 2017) and community health initiatives using professional soccer clubs as a setting for programme delivery (Robertson *et al*., 2013; Wyke *et al*., 2015; Lozano-Sufrategui *et al*., 2019; Pringle *et al*., 2021). A number of these community-based programmes, including the Football Fans in Training (FFIT), EuroFIT and Premier League Health (PLH) initiatives, are gender-sensitized programmes targeted at men, which include physical activity sessions and health education. Evaluations have shown a significant impact on weight loss, physical activity, diet and self-esteem (Wyke *et al*., 2015) and, importantly, emphasized the potential of sport, in this case football, in recruiting and maintaining participation of men in a health promotion initiative (Robertson *et al*., 2013; Geidne and Van Hoye, 2021).

A recent review of health promotion initiatives across European professional football clubs noted that most activity was oriented towards younger people (Røynesdal *et al*., 2021). This group offers a strategic return for clubs in terms of partnership and investment, thus positioning HPSC activity as corporate social responsibility (CSR) work for clubs (Lozano-Sufrategui *et al*., 2019; Pringle *et al*., 2021; Røynesdal *et al*., 2021). Approximately one-third of European professional football clubs examined offered physical activity or healthy-eating programmes for adults (Røynesdal *et al*., 2021), but there was a relative paucity of evidence concerning initiatives for older adults. Sport participation, in an age-appropriate form that includes social interaction, is attractive for older adults (Jenkin *et al*., 2021) and sports-club membership has been found to be beneficial for this group (Watts *et al*., 2017). Several UK football-club-based programmes, targeted specifically at older adults, have reported good recruitment and engagement, and positive impacts on social and psychological health, albeit with little notable impact on physical health outcomes (Bingham *et al*., 2014; Pringle *et al*., 2014; Parnell *et al*., 2015; Lozano-Sufrategui *et al*., 2017). In synthesizing findings across this literature, offering a variety of activities, an emphasis on social interaction and inclusivity, and experienced practitioners were fundamental to older adults’ engagement in programmes. Despite some progress in this area, additional research and practice linking and evaluating older adults’ experiences in health promotion initiatives within sports-club initiatives is recommended (Wolter *et al*., 2021).

### The current study

The aim of the current study was to explore the experiences of participants and service providers in the Extra Time Hub (ETH), an initiative delivered via football clubs and designed to promote physical activity, health and social connections among older adults. The English Football League Trust is the community arm of the English Football League and is responsible for advising, representing, supporting and resourcing the club community organizations (CCOs) within each of the 72 clubs in the English Football Leagues, to create stronger, healthier and more active communities (English Football League Trust, 2023). A growing body of literature has focussed on health promotion initiatives in football clubs (Pringle *et al*., 2021; Røynesdal *et al*., 2021), yet scant attention has been directed towards the experiences of older adults within these contexts. Consequently, we sought to contribute original insights to literature on health promotion initiatives in sports clubs, and the broader literature on physical activities and active ageing. Our findings can also inform the design, delivery, evaluation and promotion of analogous future initiatives.

## METHODS

### Intervention context

Supported originally by funding from Sport England and the English Football League Trust, 11 ETHs were launched by CCOs linked to football clubs across England in 2019. The ETHs are weekly social gatherings at football clubs (e.g. stadia) that seek to bring people who are retired, semi-retired or approaching retirement together to socialize and be active, with intended outcomes of the programme including improvements in participants’ physical and mental health, life satisfaction and sense of belonging within their neighbourhood and community. The football club germane to the study, located in the Midlands region of England, had a professional men’s team that competed in one of the lower tiers of the English Football League, and an amateur women’s team that competed in a national league run on a regional basis. The CCO’s premises were based at the football club’s stadium, but it delivered activities in a wide range of locations, including within the club grounds (e.g. within the stadium, adjacent football pitch) and in community venues across the city and region. Activities for the ETH at the CCO have been delivered on a weekly basis since 2019 in a football club’s: city-centre stadium and adjacent football pitch; at several community venues across the city; and via online Zoom calls, which were necessary during periods when movement restrictions were in place during the COVID-19 pandemic. The mission of the CCO is to improve the physical, mental and social wellbeing of individuals and the local community through work in several key areas, including sport and physical activity, health and mental wellbeing, and inclusion and community cohesion.

The main ETH session took place on Wednesday mornings for 2 h. During this session, which cost £3 to attend, hub members could engage in different activities, including quizzes, bingo and physical activities (e.g. bowling, table tennis). Light refreshments were also provided, giving attendees a chance to socialize before, during and after sessions. Additionally, several other activities linked to the ETH were available throughout the week, both within the football stadium grounds (e.g. seated exercise sessions, walking football) and at community venues (e.g. local parks, new age kurling) within a 15-min drive of the stadium. Some activities were free (e.g. wellbeing walks at local parks), whilst for others, a £2 fee was paid for facility hire. All sessions were delivered and facilitated by employees of the football club’s CCO. Older adults (over 55s) were recruited via club communication channels (e.g. website, match programmes, social media, contact with season-ticket holders), local media adverts, partner health services (i.e. via social prescribing) or charitable organizations for older adults, and by word of mouth.

### Research design

This study was underpinned philosophically by ontological relativism and a subjective epistemology, where data represent a co-construction of knowledge between researcher(s) and participants (Tamminen and Poucher, 2020). A semi-structured interview approach was employed, as we sought to generate rich, detailed accounts from older adults and service providers at the ETH. Aligned with our epistemological position, we recognized the importance of being reflexive about our own positions and how these shaped the construction of knowledge. All research team members had extensive qualitative research experience, including in community-based health promotion activities and/or sports clubs. In reflecting critically on our situatedness, we fully acknowledge limitations in our lived experience of participants’ lifeworlds, as most of the researchers were younger than participants, all identified as female, and were physically active.

### Procedures

Ethical approval for the study was provided by the first author’s institution’s research ethics committee. Individuals were eligible to take part in the study if they were (i) a participant in the ETH at the football club focussed upon in the study, or (ii) a staff member delivering activities at the same ETH. In October 2021–February 2022, the CCO shared the study information via email with over 150 eligible individuals who had taken part in ETH activities, with the same information shared with both staff involved in delivering the programme in May 2022. No incentive was offered for participation. In total, 10 primarily White-British (90%), older adults (female *n =* 6, male *n =* 4), who had been attending the ETH for 3–24 months, were recruited. Two staff members (female *n =* 1, male *n =* 1) took part, one of whom had been involved in delivering the programme since it commenced in 2019, and the other since 2020. Most participants attended the ETH stadium session on Wednesday mornings (*n =* 7), with the other three participants attending activities specific to older adults linked to the hub (i.e. wellbeing walks and walking football). Interviews were conducted face-to-face with the ETH participants (ETHP) from October 2021 to April 2022 (*M* length = 56 min) and online via Microsoft Teams with staff in June to July 2022 (*M* length = 32 min). During this time, all activities were being delivered in person, although some ETHPs attended activities delivered virtually during the COVID-19 pandemic. To aid contextualization and familiarization and enrich the data, the first author attended the ETH on some Wednesday mornings. Most ETHP interviews (*n =* 9) were conducted by the first author, with the remaining ETHP and both staff members interviewed by the fourth author. The semi-structured interviews explored key areas via curiosity-driven questions (Smith and Sparkes, 2016) that generated information about: how ETHPs became involved in the ETH; their perspective on the ETH as an initiative; and the meaning of taking part in the activity for them. In the case of staff members, interview discussions focussed on: the role staff played; their experiences of delivering the ETH; and challenges they faced working on the programme. Before the interviews concluded, participants were offered an opportunity to add further information. All interviews were audio-recorded and transcribed verbatim by the research team.

### Data analysis

Our analysis drew on principles for reflexive thematic analysis (Braun and Clarke, 2019, 2021), involving recursive shifts between stages. The first author led analysis of the ETHPs’ transcripts, with the fourth author leading analysis of the staff members’ transcripts. After enhancing familiarity with the dataset through reading and re-reading the transcripts, we identified meaningful segments of text and labelled these as codes. For example, the segment, ‘As soon as I go in there today, we will feel as though we are all friends’, was coded as ‘forged new friendships through the ETH’. After developing initial codes, these were reviewed and codes considered to hold shared meaning were grouped into more substantive, overarching candidate themes. For instance, the codes, ‘forged friendships’ and ‘a chance to have a conversation’, were combined within the theme ‘Friendships, conversation, and social connectivity’. Throughout our analysis, we engaged with extant literature to deepen our interpretations. The first and fourth authors worked together as ‘critical friends’ (Smith and McGannon, 2018), combining our respective analyses, challenging each other’s interpretations, and (where relevant) offering alternative interpretations, drawing on our different disciplinary backgrounds. Subsequently, the first author re-engaged with the analysis, dataset and also literature that could aid interpretations, together with critical dialogue with the second and third authors. At the conclusion of this process, we wrote up our six themes, using participant quotes to highlight their ‘voices’, and literature to demonstrate our interpretative analysis.

## FINDINGS AND DISCUSSION

### ‘The power of the badge?’ An allure for some, but not for all

Similar to past research with older adults in football-based health promotion activities [e.g. (Pringle *et al*., 2014; Parnell *et al*., 2015)], the connection with a football club provided a ‘hook’ for some ETHPs. Six ETHPs (female *n =* 3, male *n =* 3) were football supporters, with five being past or current supporters of the club. ETHP 1 initially became aware of the ETH through an online fan forum, explaining that the connection with the football club was a salient reason for joining:

I didn’t really know what it was, but anything that was with the club in the [club social area] and associated with the football club, to me, it is always going to be a bonus anyway. As I started to learn a bit about this and a bit about that, I thought ‘great’.

Likewise, from a staff perspective, the club ‘brand’ was considered important for promotion and viewed as the ‘driver’ at the outset. Although the allure of the football club was important for some, a football allegiance was not necessarily a hook for others. Similar to previous HPSC research in Ireland (Lane *et al*., 2021), the ETH successfully recruited non-sports club members, in turn providing an enhanced reach into the community. Accordingly, the affiliation with health, supported by partnerships with relevant agencies and a credible club governance model, appeared to open the gates of the football club to other older adults in the community. ETHP 7 had previously been a supporter of the football club, but only became aware of the ETH through social prescribing. When asked about how she joined the ETH, she responded:

I think it was the local social services. They sent me details about the hub, when I think I’d spoken to them about my partner’s needs and my needs and what was going on at home. So, they sent me an email about this, and I thought it would be worth a try.

Similarly, another participant reported joining through social prescribing, while the remaining three non-football club supporters joined the ETH due to partner organization connections (e.g. Age UK, army veterans club). Although these participants had limited interest in football, none stated that this limited their ability to feel included, with social inclusion aided by the lack of emphasis placed on football during activities, both by staff and other attendees. Furthermore, some felt that physically locating activities outside the football stadium widened the potential catchment area:

With the [football club community trust] coming into being, it has shown the public and those who want to take it up, it isn’t just a football club. It does take in the community. I think that as far as going on the walks and that, they do get quite a few people who do the walks in their own areas, which shows that the football club actually covers a bigger area than just this stadium. (ETHP 6)

Despite these positive reports, it was felt that many might be unaware of the ETH and that the football club connection could lead some to assume a football interest was requisite to feel welcome: ‘I am sure there are still people who might see [the crest], and then if it is [community arm of football club] with [crest], then they can’t go because they have no affiliation to the club’ (ETHP 1). Overall, these findings demonstrate that the association with a football club helped to reach individuals with an interest in football, and that relationships with local partner agencies facilitated engagement with a wider range of older adults, thus broadening the reach of the health promotion initiatives in the sports club within the community.

### ‘I don’t want anything competitive at my age, you are there to enjoy it’

One objective of the ETH is to increase the quantity of physical activity in previously ‘inactive’ and ‘active’ older adults, yet none of the ETHPs stated that increasing physical activity was a core motivation for joining. Although our study did not seek quantified physical activity data, most participants were already active to some degree prior to commencing the ETH and tended to report limited impact on the *quantity* of physical activity they undertook. In contrast to the lack of perceived impact on quantity, however, participants clearly identified the impact on the *quality* of their physical activity experiences. At the core of such quality lay hedonic aspects of physical activity, with enjoyment, fun and relaxation reported by many. Enjoyment of physical activity is associated with longer-term engagement (Williams *et al*., 2008; Allen-Collinson *et al*., 2011), and experiences of fun and enjoyment were widely discussed by participants. Most also commented positively on the unpressurized, non-competitive atmosphere involved:

It is very relaxed. You can play table-tennis, I am not any good at it, but you can just take part in things without feeling you have to be an expert. It is not competitive. It is just done purely for fun, which I think is important, I think. (ETHP 7)

Why is that important? (Interviewer)

I don’t want anything that is going to be competitive, not at my age. You are just there to enjoy it. It is just about enjoyment. (ETHP 7)

The importance of non-competitive physical activity resonates with other research on physical activity programmes [e.g. (Allen-Collinson *et al*., 2011)]. The psychological benefits of novelty (González-Cutre *et al*., 2016) and perceived variety (Dimmock *et al*., 2012) were discussed by ETHPs, and considered helpful for promoting fun and enjoyment, a point further reaffirmed by staff. Another salient element in promoting enjoyment was the importance of engaging in physical activity socially, a theme we expand on below. For instance, ETHP 1 pointed out that, ‘going for walks with four people or six people, like I have been on two already this week, I find this more enjoyable than going on my own or just me and the dog’. Alongside this, participants commented on the enjoyment of being able to choose the type, intensity and duration of (physical) activities, and being able to move in a way that suited their competence. Using self-determination theory (Deci and Ryan, 2000) as an interpretative lens, the physical activities offered, and the experiences of participants in these activities, appeared to be supportive of the basic psychological needs of competency, autonomy and relatedness. Whilst these findings offer limited evidence to suggest perceived changes in the quantity of physical activity, participants commented positively on various qualitative aspects that enriched their experiences of being physically active (see later sections).

### Friendships, conversation and social connectivity

Aligning with the English Football League Trust’s (English Football League Trust, 2022) aspirations, this theme portrays the social benefits of the ETH reported by all participants. Social isolation and loneliness are risk factors for poor health (Coyle and Dygan, 2012) and concerns about social isolation and loneliness have grown since the COVID-19 pandemic (Wu *et al*., 2020). Similar to other community-based health promotion initiatives for older adults [e.g. (Symes *et al*., 2023)], the ETH enabled participants to meet people, engage in conversation and build new friendship networks. Some, especially those who lived alone or often spent lengthy periods of time at home during their week, reported that connecting with other people at the ETH was important for addressing social isolation:

It does make me feel better, you know if you are stuck at home and down today, just to get out and talk to somebody is good. There are days when apart from talking to my partner, who hasn’t got much conversation, I don’t talk to anybody else. And I think we all need that. We all need social interaction. (ETHP 7)

Alongside addressing social isolation, participants commented on how the ETH reduced loneliness. As ETHP 5 stated: ‘It stops me from feeling lonely, and if I could encourage other people to come here and not feel lonely and be part of it, then that is great’. Likewise, staff also recognized that the ETH was about building connections and friendships rather than the activity *per se*. Meeting new people had also provided ETHPs with social support, broadened their outlook on life and developed new understanding of their community. Moreover, socially connecting with people made some more willing to converse and partake in social activities:

I think it has made me a better person. I can see that if I don’t do a lot, I can fall back into my shell a little bit, so it has definitely influenced me, because when you get there, people there probably think I talk a lot more than I do, because if I didn’t go out or just saw one person, I would be a lot quieter. (ETHP 1)

Consistent with the ‘social cure’ thread within research on social determinants of health (Haslam *et al*., 2018), our findings demonstrate the benefits of the ETH for older adults’ social relationships, social interactions and social support. More so, the social interactions at the ETH were also perceived as helpful for improving the mental health of participants, as we now elaborate.

### ‘You definitely feel a lot better in your mental health’

The ETH had become an important way to protect and promote mental health, through the enaction of cognitive and social skills, helping participants cope with life events, and enabling performance of their social roles. For some, participation helped to protect against mental ill-health: ‘I have a bit of PTSD [post-traumatic stress disorder], which this [ETH] helps me immensely with, because I don’t have to sit and think about it’ (ETHP 2). Linked to the previous theme, a salient pattern concerned the perceived mental health-enhancing impact of social interactions:

If I didn’t come, I’ll be sat at home. I’ll be looking at the wall and you’ve got things going through your head all the time, saying ‘no, no, not yet’. [When] You come here, there’s none of that because first thing you do is [say] ‘Morning. Morning everybody’, and then as soon as someone sits next to you, you start talking and everything’s gone. There’s nothing, you know, I mean there’s no depression there then, it’s all gone because someone spoke to you…Hopefully they keep it going, because if they don’t, all I’ll do is, I’ll sit at home again, do nothing. I’ve been miserable and I don’t want to do that anymore. (ETHP 4)

As portrayed here, the ETH had become an important part of this participant’s life and the thoughts of not being able to attend evoked worry. Likewise, ETHP 7 discussed feeling better after attending the ETH, explained how it helped her to cope, and outlined that because of these perceived benefits, the ETH had become a social priority in her week:

I feel better when I get home. I feel more able to cope, if you like. Yeah, it does you good. I look forward to coming. I try now to definitely keep Wednesday mornings free so that I can come here. So I say ‘I can’t do that on Wednesday because I come here’.

Alongside the mental health-enhancing benefits of social activity, several participants also discussed the perceived benefits of engaging in various cognitively stimulating tasks (e.g. quizzes, sudoku). More broadly, some felt that taking part in the ETH had boosted their confidence in social situations and their self-worth. ETHP 8, for example, had returned to walking football, 20 years after retiring:

It gives me a feeling that I have still got something to give [pause], because when you are retired, unless you find other interests, you can be cast upon the heap and feel that you have not got any worth anymore, but this is something I want to do for *me*. It gives me a buzz, it gives me a feeling that I am doing some good.

Taken together, our findings clearly demonstrate that the ETH was perceived to have a positive impact on mental health.

### The importance of pleasure

Recent critiques of physical activity health promotion have emphasized the need to shift away from dominant biomedical perspectives and an almost exclusive focus on health outcomes of physical activity (e.g. Tulle, 2017; Pullen and Malcolm, 2017; Williams *et al*., 2018), to more critically informed and diverse understandings of the meanings surrounding physical activity (e.g. Phoenix and Tulle, 2017). Consistent with such critiques, this theme demonstrates how the ETH was much more than an activity pertinent to quantifiable health-related outcomes. Although our study extends beyond solely physical activities, we draw here—at least in part—on Phoenix and Orr’s (2014) typology, to interpret the pleasures of engagement in the ETH. Many participants commented on the pleasure of habitual action (see also Phoenix and Orr, 2014), such that the ETH gave a sense of structure, direction and purpose to participants’ everyday lives, something some felt they had lost since retiring:

Most people do need to do a little bit more physical and probably mental-type activities. So, if they can come somewhere like this and they can do a bit of walking football, a bit of walking cricket, a bit of health walks and all those kind of things, it’s giving them something to look forward to. (ETHP 1)

As portrayed in the above extract, participants also reported feelings of anticipatory pleasure; thinking about the ETH elicited pleasant emotions. This was portrayed by ETHP 5, who drew attention to the pleasures of knowing that the ETH would be part of her day:

When I think ‘what day is today?’ [I think] ‘oh yeah [the Hub]’ [smiling and excited] and you get your trainers on [pointing towards shoes] and your gear [points to jacket], and I am ready. Excitement. It’s something to look forward to.

For those who engaged in the wellbeing walks, the pleasures of sensory engagement in green spaces were also discussed. ETHP 10, for example, described a walk she had just completed: ‘Today, we’ve been round here we have been looking at different birds and saying, “can you see so and so?” We discussed various flowers like there’s some white violets down there’. These pleasures of sensory embodiment resonate with other research findings on physical activity programmes (Allen-Collinson *et al*., 2011). As the group leaders and co-walkers drew attention to various aspects of the environment around them, this enriched the visual and auditory pleasures of walking, which helped to promote immersive pleasures (Phoenix and Orr, 2014). Although ETHP 3 found pleasure in the social interaction of the ETH walks, she also spoke about how conversation-free periods on the walk enabled sensory engagement with the wider ‘soundscape’ (Schafer, 1994) and the situated pleasures of the surrounding environment: ‘I love it when we go for a walk because we don’t have to talk. We could just walk, [and] listen to the birds as we are walking along’. In contrast, underscoring the importance of social interactions, the social pleasures of the ETH were regularly discussed. For some, the ETH enacted the pleasures of giving and eliciting happiness and joy in others. When asked, ‘Why has taking part in the ETH been worthwhile for you?’, ETHP 2 said:

It has given me the satisfaction of being able to sit down and talk to people. I bring the dog in and all of these, I keep saying ‘older people’, they are all the same as me, and they say, ‘can I give the dog [rubs fore finger and thumb to indicate food]?’, [and I say] ‘Yeah go on’, and I can see the joy in their face to give my dog a bit of biscuit. If that is causing them five minutes of happiness, then so be it, we will bring the dog again. That’s why I want to take part.

Here, ETHP 2 draws attention to the reciprocal pleasures derived from making others happy, further highlighting the role of the ETH as a health promotion initiative that can engender and promote pleasure among older adults.

### Going above and beyond: the pivotal role of staff

Like many community-based, health promotion programmes in football [e.g. (Bingham *et al*., 2014; Curran *et al*., 2014; Lozano-Sufrategui *et al*., 2017)], staff were reported to play a pivotal role. The friendly and welcoming demeanour of staff was noted, with several participants commending how support from staff transcended the ETH programme. As ETHP 9 noted, ‘If you have a problem outside the hub, each and every one of them would give you support if you needed it’. Paralleling past research (Lozano-Sufrategui *et al*., 2017), creating an atmosphere that fostered a sense of community and interpersonal bonds between participants was considered important for staff and ETHPs. For some, the care and support from the ETH staff had improved their state of mind:

They always have a smile on their face. They always have time for you. You can talk to them about your own problems, so without any doubt, you definitely feel a lot better in your mental health than if you hadn’t come out. (ETHP 4)

The role of supportive staff resonates with other health promotion programmes, such as exercise referral, where knowledgeable, reassuring and supportive instructors were considered invaluable (Moore *et al*., 2013). The importance of such qualities was noted by staff during the COVID-19 pandemic, when lockdown restrictions necessitated adaptation to services. During this period, befriending calls, grocery shopping and online meet-ups were organized for ETH members and season-ticket holders. Reaching out to the ETH members engendered realization for the ETH staff:

It really made us realise the need of people very locally to us, when we’re finding all these people who were so lonely and they wanted someone to talk to every week and we realised actually, you know, there’s people that had no one, no family, no friends in [place]. They couldn’t even go and do their shopping, we realised kind of more what our role could be. (Staff 1)

The support that staff provided during the lockdown periods was appreciated by ETHPs who engaged with the club at this time. Connecting with ETH members during lockdown provided a vital support network to many, with the ETH subsequently increasing its membership by 300% upon returning to face-to-face activities. Staff spoke about then being focussed on how they could develop the existing service and build a sustainable ETH.

## CONCLUDING THOUGHTS

The study explored the experiences of participants and service providers of a settings-based health promotion initiative for older adults, delivered by the community arm of an English League Football club. Our research demonstrates how the brand of a sports club, and in this case a football club, can help attract older adults to partake in health-related activities. Furthermore, the ETH in the current study was also successful in widening participation, to include older adults with no allegiance to football, through effective referral mechanisms and promotion of the ETH with local partner agencies. Consequently, this emphasizes the importance of dialogue between health services and sports clubs promoting health initiatives to ensure that awareness is raised about the activities on offer. Additionally, it demonstrates the potential for sports clubs to widen engagement with the local community through an affiliation with health promotion (Lane *et al*., 2021) for, in this case, older adults.

Consistent with previous studies, we show that the sports club can be a valuable setting to promote health, with our research adding fresh insights to the small but growing corpus of evidence specifically on health-promoting initiatives for older adults in football clubs. Accordingly, our study demonstrates the potential for perceived improvements in mental health, quality of physical activity experiences and social connectedness through engagement in the ETH. Whilst sport-related groups for older adults can have social and health benefits, our findings reinforce the importance of the role played by experienced staff in enabling this (Wolter *et al*., 2021). Although the long-term English Football League Trust model was intended to create a self-sustaining programme (i.e. members could be responsible for programme delivery on a voluntary basis), given the clear importance of staff for the experiences of the ETHPs, further consideration as to how a self-sustaining programme could be implemented (effectively) is warranted.

Theoretically, our study also makes a contribution to understandings of pleasure in older adulthood (Allen-Collinson *et al*., 2011; Phoenix and Orr, 2014; Tulle, 2017) and highlights the potential to diversify understandings of physical activity within health promotion initiatives in sports clubs. Drawing on elements of Phoenix and Orr’s (Phoenix and Orr, 2014) typology, we illustrated how ETHPs sourced pleasure from sensory experiences, habitual action and immersion through activities in the ETH. It should also be noted that the pleasures described by the ETHPs were not limited solely to physical activity, thus demonstrating a form of conceptual generalizability (Smith, 2018) with respect to Phoenix and Orr’s (Phoenix and Orr, 2014) typology of pleasures. Currently, the physical activity guidelines for older adults (Department of Health and Social Care, 2019) continue to emphasize the health benefits of physical activity, while sport-for-health promotional messaging tends to focus only on the health outcomes derived from participating in such activities (McFadyen *et al*., 2018; Geidne *et al*., 2019). Based on our findings, and, specifically, the centrality of pleasures to the experiences of older adults, future efforts to promote physical activity and health-promoting activities in sports clubs to this particular population could draw on co-production methods (Smith *et al*., 2023) to identify the messaging most suited to engaging older adults with health promotion initiatives within sports clubs. For instance, the possibilities of co-production to amplify pleasures are highlighted in recent research with disabled people, where unanimous support for emphasizing the potential enjoyment of physical activity was obtained (Smith and Wightman, 2021). Therefore, attempts to promote physical activity in older adults through health promotion initiatives in sports clubs should embrace hedonic perspectives rather than rely on biomedical perspectives, ensuring that the pleasures of physical activity are emphasized.

Overall, our findings contribute to understandings of settings-based health promotion activities in sports clubs, particularly in relation to older adults in football clubs. Nevertheless, we acknowledge that the study focussed on understanding the perspectives of a small number of older adults and staff involved in the ETH at one English League Football club. Thus, although our findings might hold the potential for naturalistic generalizability (i.e. through resonating with wider populations; Smith, 2018), further research is warranted to understand the experiences of individuals in health promotion initiatives delivered for older adults in other sports and physical activities, countries and cultures. Findings of the current study demonstrate that sports clubs that have staff members to organize and deliver activities, as well as links with partner agencies, have the potential to contribute positively to the lives of older adults through health promotion initiatives delivered within their club. Nevertheless, other sporting organizations have embraced networks of volunteers, specifically activating relevant skillsets, to support the delivery of health promotion in sports clubs, often with the support of health and sport agencies (Lane *et al*., 2021). The application of this volunteer-driven model to health promotion initiatives for older adults delivered in voluntary, grassroots sport clubs warrants further examination, as this could have far-reaching benefits for communities. Ultimately, with the increasing life expectancy of much of the global population, expanding the evidence base on settings-based health promotion initiatives for older adults within sports clubs more widely will better equip clubs to capitalize on the potential to utilize this setting as a vehicle to promote both pleasure and health-related outcomes.
